# Prognostic relevance of elevated pulmonary arterial pressure assessed non-invasively: Analysis in a large patient cohort with invasive measurements in near temporal proximity

**DOI:** 10.1371/journal.pone.0191206

**Published:** 2018-01-19

**Authors:** Sebastian Greiner, Andreas Jud, Matthias Aurich, Christoph Geisenberger, Lorenz Uhlmann, Thomas Hilbel, Meinhard Kieser, Hugo A. Katus, Derliz Mereles

**Affiliations:** 1 Department of Cardiology, Angiology and Pneumology, University of Heidelberg, Heidelberg, Germany; 2 Division of Experimental Neurosurgery, University of Heidelberg, Heidelberg, Germany; 3 Institute of Medical Biometry and Informatics, University of Heidelberg, Heidelberg, Germany; Scuola Superiore Sant'Anna, ITALY

## Abstract

**Background:**

The clinical relevance of non-invasively derived pulmonary arterial pressure (PAP) by Doppler echocardiography (DE) has been questioned in the past. However, transthoracic echocardiography is used as a cornerstone examination for patients with dyspnea and suspected pulmonary hypertension (PH). This study aimed to evaluate the prognostic value of non-invasive assessed PAP in a large population of patients with known or suspected cardiopulmonary disease.

**Methods:**

The analyses are based on data of patients of a tertiary cardiology center that received right heart catheterization (RHC) as well as non-invasively assessed PAP by DE within five days, and includes serological and clinical parameters in a retrospective follow-up for up to eight years.

**Results:**

Of 1,237 patients, clinical follow-up was possible in 1,038 patients who were included in the statistical analysis. The mean-follow up time was 1,002 days. The composite endpoint of heart transplantation (HTx) or death occurred in n = 308 patients. Elevated PAP measured non-invasively as well as invasively had significant prognostic impact (hazard ratio (HR) 2.32; 95% confidence interval (CI) 1.78–3.04; χ^2^ = 37.9; p<0.001 versus HR 2.84; 95%CI 2.11–3.82; χ^2^ = 51.9; p<0.001, respectively). By multivariate analysis, NYHA functional class, N-terminal pro-brain natriuretic peptide, cardiac troponin T, left ventricular ejection fraction, and right ventricular dysfunction remained independently predictive. Incremental prognostic information in a multimodal approach was highly relevant.

**Conclusions:**

In this comprehensive study, elevated pulmonary arterial pressure measured by DE offers similar prognostic information on survival or need for HTx as right heart catheterization. Furthermore, the addition of functional capacity and serological biomarkers delivered incremental prognostic information.

## Introduction

Pulmonary hypertension (PH) is generally recognized as an important predictor of morbidity and mortality in patients with cardiopulmonary diseases [1,2]. PH must be assessed invasively for appropriate targeted therapy. However, standard transthoracic echocardiography including Doppler measurements is used in daily practice as a screening method to identify patients before referral to right heart catheterization (RHC) [3–6]. The guidelines for the diagnosis and treatment of PH incorporate Doppler echocardiography (DE) for risk stratification [7]. The accuracy of DE has been analyzed by our group in 2014 in a large patient cohort, which included all entities of PH, and detailed information on the technical feasibility and diagnostic reliability. In addition, limitations of this method under real-life conditions were provided by these analyses [8]. However, controversial discussions on the clinical relevance of non-invasive measurement of pulmonary arterial pressure (PAP) in individual patients persist [9–11].

The prognostic data of invasive as well as non-invasive PAP measurements in patients with PH due to left heart disease (LHD, PH group 2) is limited, although they represent the majority of patients with PH [7]. The largest, unselected datasets of patients with PH are provided by observational population studies. Those community studies include up to 10,314 individuals, but lack invasive measurements for confirmation of PH [12,13]. Recent studies describe elevated PAP as a risk factor for adverse outcome in selected patient cohorts as acute and chronic left heart failure, heart failure with preserved ejection fraction (HFpEF) and valvular heart disease, but here again, data from RHC as a reference is lacking [14–17]. Clinical trials that deliver prognostic information of PAP measurement including RHC are focused predominantly on patients with pulmonary arterial hypertension (PAH), and therefore, have highly selected and mostly small cohorts [7,18–22]. The largest survival analysis on 2,749 patients with precapillary PH is based on the REVEAL registry, but it does not provide information on non-invasive PAP measurements [23,24]. Furthermore, until now no meta-analyses of survival studies on patients with PH including DE and RHC are available.

The aim of this study was to assess the prognostic value of non-invasive PAP assessment for the individual patient based on a large study population of patients with suspected PH of any cause, in direct comparison to invasive PAP measurement by timely matched RHC.

## Material and methods

### Study protocol

**Fig 1:**
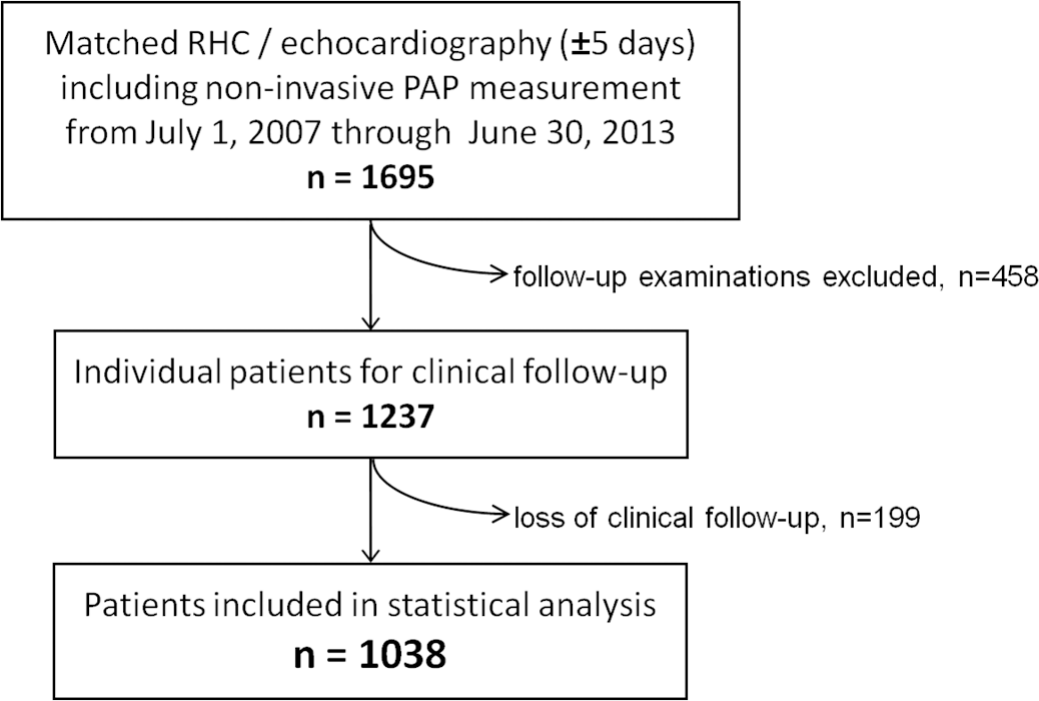
Study protocol. Flow chart with inclusion criteria from catheter and echocardiography databases, identification of individual patients, and exclusion due to loss of clinical follow-up. **Abbreviation:** RHC right heart catheterization.

The study is based on data of patients that received RHC and DE examinations within five days at the Cardiologic Department of the University Hospital of Heidelberg between July 1, 2006 and June 30, 2013 (Fig 1). Analyses of follow-up of the screened patients were carried out until June 30, 2015 after approval by the Ethics Committee of the University of Heidelberg, in accordance with the current version of the Declaration of Helsinki, and with informed consent of the patients as stated verbally.

All patients included in the study had appropriate clinical indications for RHC (Table 1). We identified 1,695 examinations including DE with systolic PAP measurement within 5 days before or after RHC in our heart catheter and echolab databases (55% within one day). Four hundred fifty-eight examinations were follow-up examinations; thus 1,237 individual patients were included in the clinical follow-up. The follow-up was obtained by review of the digital clinical records and a telephone interview with the patient, a relative, or the family physician doctor. One hundred ninety-nine patients (16%) were lost to follow-up. Although the proportion of men was is slightly smaller in this group and patients were four years older in mean age, body baseline characteristics, serologic parameters, functional state by New York Heart Association (NYHA) class as well as ratios of cardiopulmonary disease subgroups did not differ significantly to the patients included in the analyses (S1 Tables). There were no other reasons for exclusion. The final study population consisted of 660 men and 378 women.

**Table 1:** Clinical characteristics of the study population.

Parameter	all	survivors	non-survivors	p-value
	n = 1,038	n = 730	/HTx, n = 308	
Age, y	66 ± 15	66 ± 15	65 ± 15	0.35
Males, n (%)	660 (64)	439 (60)	221 (72)	<0.0001
Height, cm	171 ± 10	171 ± 10	172 ± 9	0.69
Weight, kg	79 ± 17	80 ± 17	77 ± 17	0.17
BMI, kg/m^2^	27 ± 5	27 ± 5	26 ± 5	0.06
BSA, m^2^	1.9 ± 0.2	1.9 ± 0.2	1.9 ± 0.2	0.33
**Clinical classification, n (%)**				
NYHA functional class I	84 (8)	78 (11)	6 (2)	<0.0001
NYHA functional class II	311 (30)	257 (35)	54 (18)	
NYHA functional class III	533 (51)	342 (47)	191 (62)	
NYHA functional class IV	110 (11)	53 (7)	57 (18)	
eGFR, mL/min/1.73m^2^	66 ± 27	70 ± 26	56 ± 28	<0.0001
NT-proBNP, pg/mL^a^	2889 (847;6731)	1985 (567;5388)	4915 (2572;10143)	<0.0001
cTnT, ng/L^b^	24 (10;59)	20 (10;50)	41 (20;86)	<0.0001
**Indications for heart catheterization, n (%)**				
Known or suspected CMP	261 (25)	164 (22)	97 (32)	0.002
Evaluation valve disease				
-Aortic valve disease	186 (18)	158 (22)	28 (9)	<0.0001
-Mitral valve disease	82 (8)	78 (11)	4 (1)	<0.0001
-Tricuspid valve disease	6 (1)	5 (1)	1 (0,3)	0.68
Known or suspected IHD	315 (30)	199 (27)	116 (38)	0.001
Known or suspected precapillary PH	82 (8)	58 (8)	24 (8)	0.99
Other miscellaneous indications^c^	106 (10)	68 (9)	38 (12)	0.60

^a^assessed in n = 643

^b^assessed in n = 780

^c^primarily cardiac amyloidosis.

**Abbreviations:** HTx heart transplantation, BMI body mass index, BSA body surface area, NYHA New York Heart Association, eGFR estimated glomerular filtration rate, NT-proBNP N-terminal pro brain natriuretic peptide, cTnT cardiac troponin T, CMP cardiomyopathy, IHD ischemic heart disease, PH pulmonary hypertension

### Right heart catheterization

Clinical indications for heart catheterization are given in Table 1. As specified in our previous study [8], a femoral or jugular venous approach with local anaesthesia was used for RHC. Cardiac output (CO) and cardiac index were calculated using the Fick method. Measurement of PAP, pulmonary capillary wedge pressure (PCWP), and right ventricular and right atrial pressures (RAP) were performed during breath hold after normal expiration over at least three heart cycles. Pressure measurements were conducted several times, and zero-level adjustment at the level of the right atrium was checked repeatedly for consistent results. Mean PAP was calculated by integration of the pressure curve by Metek software (Metek GmbH, Roetgen, Germany) and was considered as elevated per definition if mean PAP (mPAP) was 25 mm Hg or higher.^7^ Pulmonary vascular resistance (PVR) was derived from PVR = (mPAP—PCWP)/CO.

### Transthoracic echocardiography

Echocardiographic examinations were performed on commercially available ultrasound systems (Vivid S5, Vivid i, Vivid 7, and Vivid E9 GE Healthcare Vingmed, Trondheim, Norway and ie33, Philips, Eindhoven, The Netherlands) according to the guidelines of the American Society of Echocardiography [5,25]. As described in our previous study [8], images were obtained in the left lateral position for parasternal and apical views and the supine position for subxiphoid views using 1.5 to 4.6 MHz phased-array transducers. The examination included standard two-dimensional (2D) echocardiography for anatomic imaging and Doppler echocardiography for the assessment of velocities. Doppler measurements were carried out over three heart cycles during passive expiration.

Non-invasive assessment of pulmonary artery systolic pressures (sPAP) was achieved by measurement of the right ventricular systolic pressure gradient (RVSP) and adding the estimated RAP as described previously [8,26]. RVSP was derived from the peak systolic velocity of the tricuspid regurgitation obtained with continuous-wave (CW) Doppler using the modified Bernoulli equation: pressure gradient (PG) = 4^x^v_max_^2^. A sPAP of 36 mm Hg and higher was considered as elevated based on the analysis of our previous study with 1,695 datasets.^8^ RV function was analyzed as documented in the echocardiography report by the examiners in 845 patients (81%). Quantitative measurements of surrogate parameters for RV function (e.g. TAPSE, Tei index, tissue Doppler velocities) were documented in 251 reports (24%), supporting the examiners’ qualitative assessment, but were not included in the statistical analyses. Moreover, all data were taken up as documented, and no secondary measurements were conducted to avoid a bias of the statistical analyses.

### Statistical analyses

Statistical analyses were performed using SPSS version 22 (IBM Corporation, Armonk, NY, US) and R including the packages mice, mitools, survival, Rcpp, lattice, and xtable (R Foundation for Statistical Computing, Vienna, Austria; www.R-project.org). In case of normal distribution, continuous data are expressed as mean and standard deviation (SD), or otherwise as median with 25% and 75% percentiles. For categorical data absolute and relative frequencies are provided. Differences between survivors and non-survivors/HTx patients were analyzed by the Student’s t-test (unpaired, two-tailed), the Mann-Whitney-U-test, or the chi square test, as appropriate. Receiver operating characteristics (ROC) were calculated to define optimal cut-off values for dichotomous analyses of each parameter in the following steps. Kaplan-Meier curves are used to show the percentage of event-free survival for up to eight years. Univariate and multivariate regression analyses by Cox proportional hazards were conducted to identify predictive parameters, including a comprehensive statistical approach with multiple imputation of missing parameters for multivariate analyses (1,971 of 18,684 in total, 10.5%; PAP measurements: n = 0 missing), as well as sensitivity analyses and validation of consistency by a complete case analysis [27].

## Results

### Characteristics of the study population

Based on the consecutive acquisition of echocardiographic examinations over a time period of six years, 1,237 patients with RHC and echocardiography in near temporal proximity, including non-invasive PAP measurement by DE, were identified from our heart catheter and echocardiography databases. Of these, 1,038 patients received a clinical follow-up (Fig 1). The mean age of patients was 66±15 years. The study population contained 660 men (64%). PH was diagnosed invasively by mean PAP ≥ 25 mm Hg in 699 patients (67%). The overall study population presented dyspnea to a high degree, with 51% of patients in NYHA functional class III (Table 1).

Non-survivors and patients who received HTx were more symptomatic than survivors (80% NYHA classes III and IV). The proportion of men was significantly higher among the diseased or transplanted patients. The serologic cardiac markers NT-proBNP and cTnT as well as renal function (eGFR) differed significantly between survivors and non-survivors/HTx (Table 1). All hemodynamic parameters were able to differentiate between the groups of patients with or without event. PAP measurements, PVR, RAP, PCWP, and echocardiographic parameters showed highest significance (p<0.0001 each). PH was predominant and more severe in non-survivors and transplanted patients (Table 2).

**Table 2:** Hemodynamic characteristics of the study population.

Parameter	all	survivors	non-survivors	p-value	
	n = 1,038	n = 730	/HTx, n = 308		
**Heart catheterization**					
sBP, mm Hg	130 ± 29	137 ± 28	116 ± 28	<0.0001	
dBP, mm Hg	66 ± 12	68 ± 12	62 ± 12	<0.0001	
CO, mL/min	4.5 ± 1.5	4.7 ± 1.5	4.1 ± 1.6	<0.0001	
CI, mL/min/m^2^	2.4 ± 0.8	2.5 ± 0.7	2.2 ± 0.9	<0.0001	
PVR, dyn^x^s^x^cm^-5^	165 (105;257)	145 (93;226)	213 (136;311)	<0.0001	
sPAP, mm Hg	46.0 ± 16.3	43.4 ± 15.8	52.1 ± 16.1	<0.0001	
mPAP, mm Hg	30.6 ± 11.0	28.8 ± 10.6	34.9 ± 10.6	<0.0001	
RAP, mm Hg	11.3 ± 5.5	10.4 ± 5.1	13.5 ± 5.8	<0.0001	
PCWP, mm Hg	20.2 ± 7.8	18.9 ± 7.5	23.5 ± 8.5	<0.0001	
**PH prevalence** (mPAP≥25mmHg)					
PH excluded, n (%)	339 (33)	286 (39)	53 (17)	<0.0001	
PH, n (%)	699 (67)	444 (61)	255 (83)	<0.0001	
**Echocardiography**					
sPAP, mm Hg	44.1 ± 16.5	41.6 ± 15.9	49.9 ± 16.6	<0.0001	
RAP, mm Hg	10.9 ± 6.7	9.8 ± 6.3	13.7 ± 6.9	<0.0001	
LV-EF, %	40 ± 17	44 ± 16	31 ± 16	<0.0001	
RV dysfunction, n (%)	456 (44)	270 (37)	186 (60)	<0.0001	
**PH indicated by DE** (sPAP≥36mmHg)	657 (63)	418 (57)	239 (78)	<0.0001	

**Abbreviations:** HTx heart transplantation, sBP systolic blood pressure, dBP diastolic blood pressure, CO cardiac output, CI cardiac index, PVR pulmonary vascular resistance, sPAP systolic pulmonary arterial pressure, mPAP mean pulmonary arterial pressure, RAP right atrial pressure, PCWP pulmonary capillary wedge pressure, PH pulmonary hypertension, LV-EF left ventricular ejection fraction, RV right ventricular, DE Doppler echocardiography

Patients with precapillary PH as defined by mean PAP ≥ 25 mm Hg and PCWP ≤ 15 mm Hg were a subgroup in this study population (n = 82). They received targeted therapy according to the current guidelines, including vasodilatory treatment, diuretic therapy, oxygen support at home, and anticoagulation, if indicated [7]. Patients with left heart disease were treated according to the underlying cardiac disease and received surgical or interventional valve repair or coronary angioplasty including coronary stenting, as well as guideline-concordant heart failure therapy by angiotensin-converting-enzyme inhibitors, beta blockers, aldosterone inhibitors and diuretic medication, implanted cardioverter-defibrillator and cardiac resynchronization therapy, up to catecholamine therapy, ventricular assist device and heart transplantation (HTx), as appropriate.

### Survival analysis

The composite endpoint of death or HTx occurred in 308 patients (n = 237 and n = 71, respectively). The mean follow-up time was 1,002 days.

**Fig 2:**
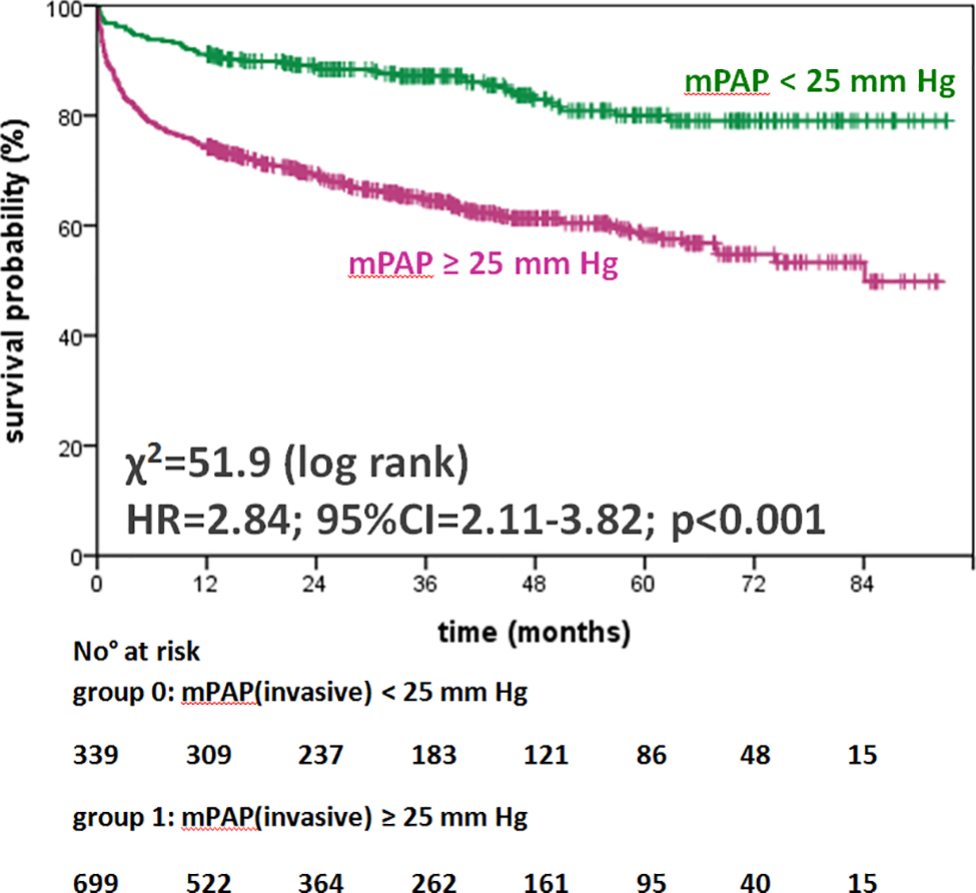
Survival of patients with or without PH as diagnosed by RHC visualized by Kaplan-Meier curves. Abbreviations: PH pulmonary hypertension, RHC right heart catheterization, mPAP mean pulmonary arterial pressure, HR hazard ratio, 95%CI 95% confidence interval.

**Fig 3:**
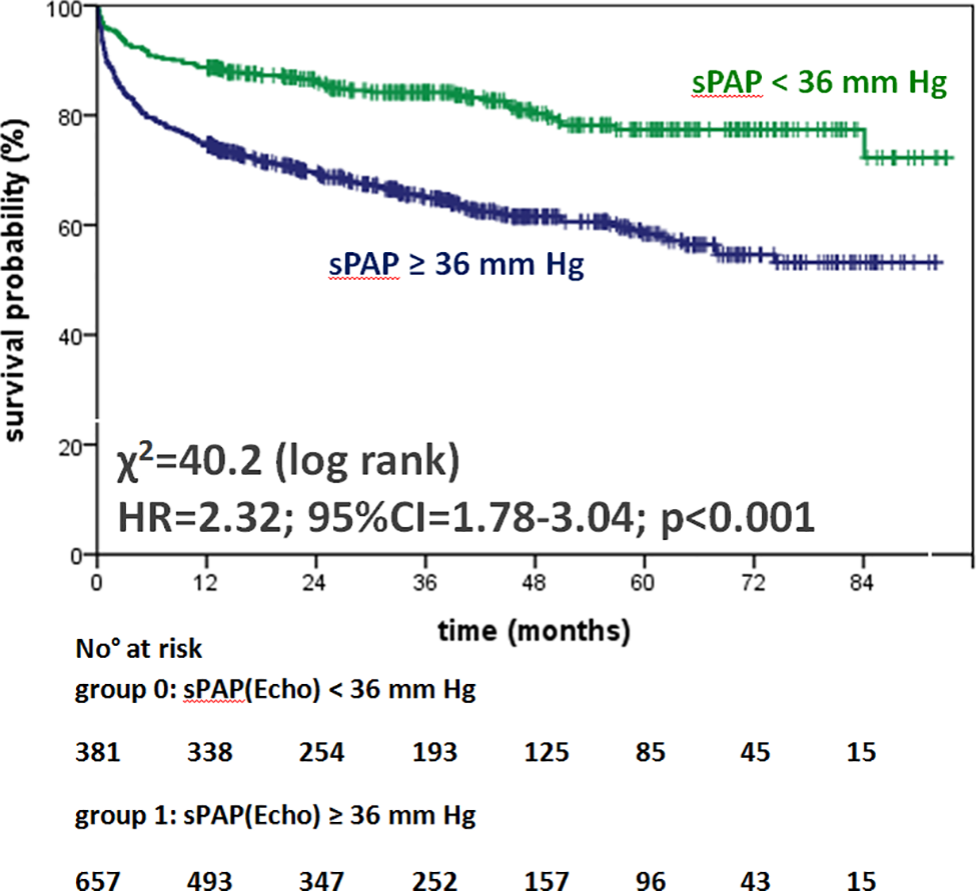
Survival of patients with or without PH as suggested by Doppler echocardiography visualized by Kaplan-Meier curves. **Abbreviations:** PH pulmonary hypertension, sPAP systolic pulmonary arterial pressure, HR hazard ratio, 95%CI 95% confidence interval.

**Fig 4:**
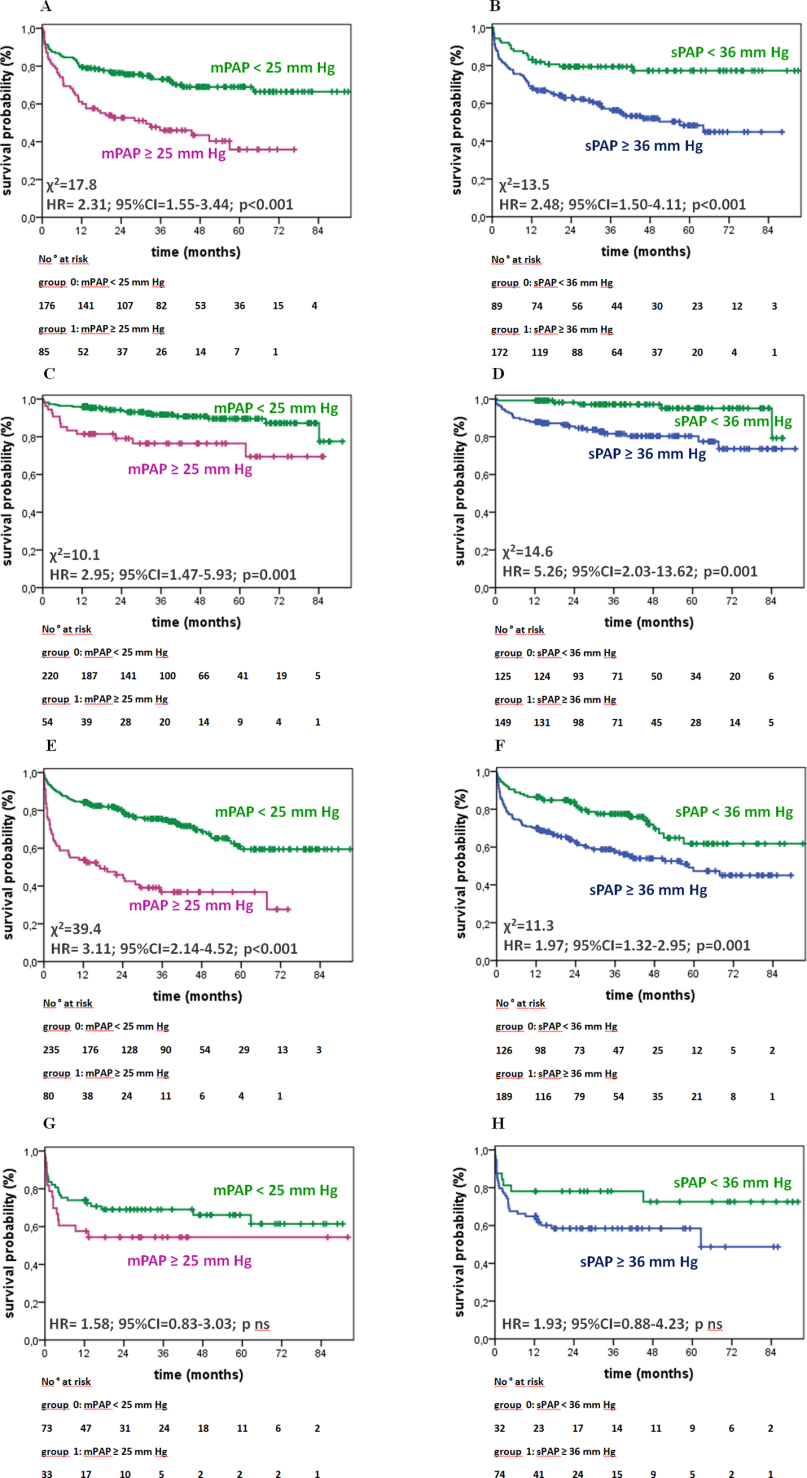
**Outcome differentiated by PAP for subgroup of patients:** left ventricular cardiomyopathy (CMP):A,B; valvular heart disease: C,D; ischemic heart disease (IHD): E,F and rare cardiac diseases: G;H. Invasive measurements by RHC (A,C,E,G) are compared to non-invasively assessment by DE (B,D,F,G). **Abbreviations:** m/sPAP mean/systolic pulmonary arterial pressure, HR hazard ratio, 95%CI 95% confidence interval, ns not significant.

**Fig 5:**
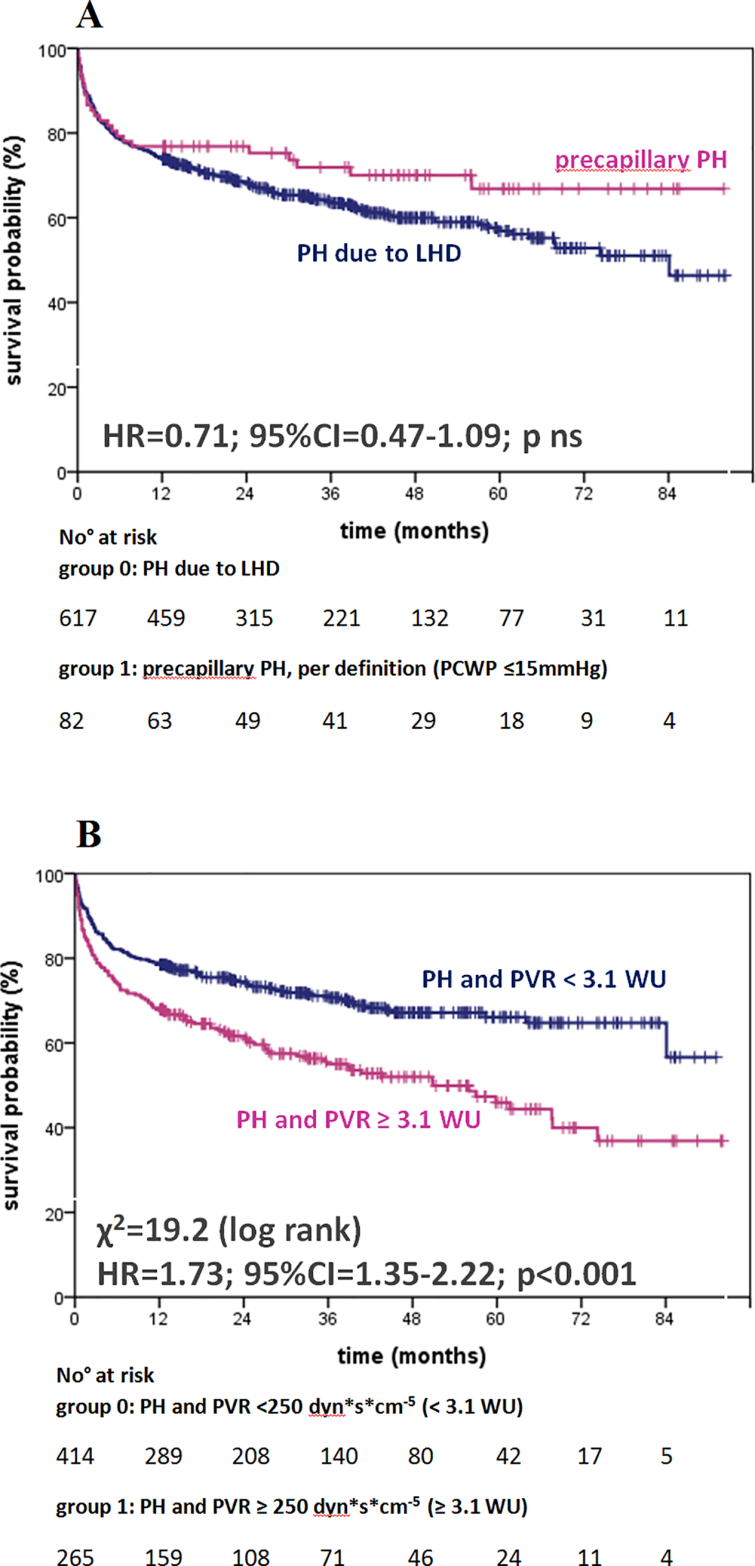
**Kaplan-Meier curves for survival of patients with PH due to LHD or precapillary PH** defined by RHC according to mPAP and PCWP (A) or elevated PVR (≥ 250 dyn^x^s^x^cm^-5^ or 3.1 WU, B). **Abbreviations:** PH pulmonary hypertension, LHD left heart disease, RHC right heart catheterization, PAP pulmonary arterial pressure, mPAP mean pulmonary arterial pressure, PVR pulmonary vascular resistance, WU Wood Units, HR hazard ratio, 95%CI 95% confidence interval, ns not significant.

Table 3 shows the unadjusted, univariate survival analyses by Cox proportional regression and χ^2^ obtained by the log rank test for each parameter: invasively (mPAP ≥ 25 mm Hg) as well as non-invasively (sPAP ≥ 36 mm Hg) elevated PAP had significant and comparable prognostic impact (hazard ratio (HR) 2.84; 95% confidence interval (CI) 2.11–3,82; χ^2^ = 51.9; p<0.001 vs. HR 2.32; 95% CI 1.78–3.04; χ^2^ = 37.9; p<0.001; Figs 2 and 3). RAP and other hemodynamic parameters such as PVR, cardiac index (CI), and left ventricular enddiastolic pressure (LVEDP), echocardiographic parameters (e.g. estimated RAP, RV diameter, RV function, and LV-EF) as well as male sex and serological parameters including cTnT, NT-proBNP and renal function (eGFR) were associated significantly with increased mortality (Table 3, S5 Fig). Fig 4 panels A to H show the outcome of patients according to elevated PAP by RHC (mPAP ≥ 25 mm Hg) or DE (sPAP ≥ 36 mmHg) grouped for the patients’ primary diagnosis. For patients with invasively diagnosed PH, the comparison to normal PAP was not possible per definition. In further analyses concerning PH, the patients with invasively defined PH (n = 699) showed no significant difference in survival between patients with PH due to left heart failure (n = 617) and those with precapillary PH (n = 82), whereas stratification by elevated PVR (≥3.1 WU) revealed significantly worse outcome (Fig 5). Survival of patients with PH stratified by sex is given in the supplemental material (S1, S2 and S3 Figs).

**Table 3:** Univariate survival analysis by Cox proportional regression.

Parameter	cut-off	HR	95% CI		p-value	χ^2^
Age	> 65 y	**0.91**	0.71	1.15	0.42	**-**
Sex	male	**1.52**	1.19	1.95	0.001	**11.2**
BMI	> 30 kg/m2	**1.50**	0.92	2.45	0.11	**-**
NYHA functional class	> II	**3.11**	2.34	4.12	< 0.001	**68.7**
NT-proBNP	> 2570 pg/mL	**3.57**	2.58	4.95	< 0.001	**66.5**
cTnT	≥ 14 ng/L	**2.93**	2.06	4.15	< 0.001	**39.7**
eGFR	< 45 mL/min	**3.02**	2.32	3.93	< 0.001	**75.2**
mPAP (invasive)	≥ 25 mmHg	**2.84**	2.11	3.82	<0.001	**51.9**
sPAP (echo)	≥ 36 mmHg	**2.32**	1.78	3.04	<0.001	**40.2**
RAP (invasive)	≥ 10 mmHg	**2.21**	1.72	2.84	<0.001	**40.3**
RAP (echo)	≥ 15 mmHg	**2.47**	1.95	3.12	<0.001	**61.4**
RV dysfunction (echo)	yes	**3.75**	2.76	5.11	<0.001	**81.5**
RV-EDD (echo)	> 32mm	**2.58**	1.89	3.53	<0.001	**30.8**
Precapillary PH (invasive)	mPAP≥25&PCWP≤15	**0.71**	0.47	1.09	0.12	**-**
PVR (invasive)	> 250 dynxsxcm-5	**2.42**	1.93	3.05	<0.001	**61.5**
LVEDP (invasive)	≥ 20 mmHg	**1.67**	1.19	2.32	0.003	**9.2**
CI (invasive)	< 2.0 L/min/m^2^	**2.18**	1.74	2.75	<0.001	**46.7**
LV-EF (echo)	< 40%	**3.23**	2.47	4.24	<0.001	**80.9**

**Abbreviations:** HR hazard ratio, 95%CI 95% confidence interval, BMI body mass index, NYHA New York Heart Association, NT-proBNP N-terminal pro brain natriuretic peptide, cTnT cardiac troponin T, eGFR estimated glomerular filtration rate, mPAP mean pulmonary arterial pressure, sPAP systolic pulmonary arterial pressure, RAP right atrial pressure, RV-EDD right ventricular end-diastolic diameter, PH pulmonary hypertension, PCWP pulmonary capillary wedge pressure, PVR pulmonary vascular resistance, LV-EDP left ventricular end-diastolic pressure, CI cardiac index, LV-EF left ventricular ejection fraction.

### Independent predictors of mortality or heart transplantation

By multivariate analysis, patients' functional capacity defined by NYHA functional classification, NT-proBNP, cTnT, LV-EF and RV function were independently predictive, whereas all hemodynamic parameters were dependent predictors (Table 4). Sensitivity analyses in the complete case datasets of 395 patients showed consistent results (data not shown).

**Table 4:** Multivariate analysis for independent predictive parameters.

Parameter	HR	95% CI	p-value
NYHA functional classes III/IV	**1.92**	1.42–2.58	<0.001
NT-proBNP (≥ 2570 pg/mL)	**1.68**	1.20–2.34	0.003
cTnT (≥ 14 ng/L)	**1.94**	1.38–2.74	<0.001
LV-EF (echo, <40%)	**1.87**	1.41–2.47	<0.001
RV dysfunction (echo)	**1.66**	1.19–2.33	0.004

**Abbreviations:** HR hazard ratio, 95%CI 95% confidence interval, NYHA New York Heart Association, NT-proBNP N-terminal pro brain natriuretic peptide, cTnT cardiac troponine T, LV-EF left ventricular ejection fraction, RV right ventricular

**Fig 6:**
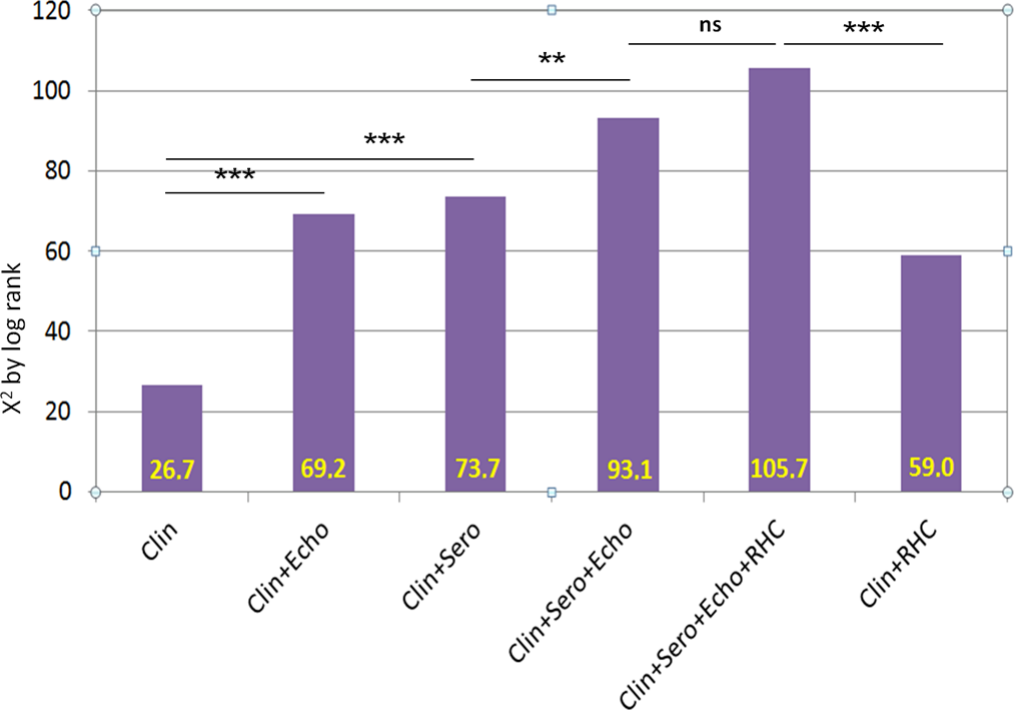
Incremental predictive information for survival of multimodal clinical settings based on complete-case data (n = 395). **Abbreviations:** Clin: clinical assessment (age, sex, NYHA functional class), Clin+Echo: clinical assessment and transthoracic (Doppler) echocardiography (LV-EF, RV dysfunction, sPAP, RAP), Clin+Sero: clinical assessment and cardiac serological parameters (NT-proBNP, cTnT); Clin+Sero+Echo: clinical assessment, cardiac serological parameters and transthoracic echocardiography combined; Clin+Sero+Echo+RHC: Non-invasive diagnostics and RHC (CI, mPAP, RAP) combined; Clin+RHC: clinical assessment and RHC (CI, mPAP, RAP) combined. **p<0.001. NYHA New York Heart Association, LV-EF left ventricular ejection fraction, sPAP systolic pulmonary arterial pressure, RAP right atrial pressure, NT-proBNP N-terminal pro brain natriuretic peptide, cTnT cardiac troponin T, CI cardiac index, mPAP mean pulmonary arterial pressure, RHC right heart catheterization.

By secondary analyses, RAP, mPAP and sPAP showed dependencies primarily to NYHA functional class, NT-proBNP, and RV dysfunction. In addition, the diagnostic approaches on patients that reflect different clinical settings with combination of clinical, serological, echocardiographic and/or invasive parameters were tested on the basis of the complete-case data and revealed the incremental prognostic information in a multimodal approach (Fig 6).

## Discussion

Adding to published data currently available [6,12–24], this study provides the statistical basis to clarify the prognostic relevance of non-invasive PAP measurements for patients with cardiopulmonary diseases in daily clinical practice: PAP measurements by DE or RHC show similar predictive value, and the combination with NYHA functional class as well as serological biomarkers adds significant predictive information.

### Technical aspects of PAP assessment

As every diagnostic tool, non-invasive PAP measurement by DE has methodical limitations that are analyzed and described in detail in our previous analysis [8], and has been confirmed in a large cohort of patients with PAH in 2016 [28]. Comparable to RHC, methodical pitfalls have to be recognized and avoided. This includes correct assessment of RAP which contributes an important part to the non-invasive assessment of systolic PAP and can be derived with high correlation to invasive measurements as well [8,29].

### Confounders for outcome in this unselected study population

This study population is representative for a tertiary cardiologic center, especially in context of the patients' primary diagnoses and indications for RHC (Table 1). The presented data were obtained systematically from examinations over a long time period of six years, without artificial selection bias: exclusion of patients was only due to loss of follow-up; there was no interaction between RHC and echocardiographic measurements as the examinations took place independently of each other and were conducted by different examiners. Nevertheless, RHC and echocardiography were in close temporal proximity within five days (55% within 24 hours).

The analyzed study population has a majority of men in the group of non-survivors/HTx. Male sex is a known cardiovascular risk factor associated with coronary heart disease and cardiovascular death [30]. In the present study, men with PH showed a significantly reduced survival compared to men without PH when assessed by RHC (men: HR 3.03, 95% CI 2.10–4.37 versus women: HR 2.33, 95% CI 1.4–3.89, p<0.001 each; S1 Fig), involving a comparable proportion of ischemic heart disease in both groups (33% vs. 32%). The subgroup with precapillary PH (n = 82) shows a non-significant trend to better survival under treatment when compared to patients with PH due to LHD (Fig 5A) that has to be differentiated by sex: 19 of 52 men with precapillary PH died during the follow-up time, but only 2 of 30 women did not survive (S2 Fig). Accordingly, there is a significant sex-related effect on mortality in this selected subgroup with precapillary PH under treatment. This finding is concordant with analyses indicating that women with precapillary PH may benefit more from vasodilator therapy than men, depending on the etiology of precapillary PH [31]. Summarized, compensatory mechanisms in patients with PH due to LHD and precapillary PH, as well as response to therapy may be related to sex, and a sex-specific bias cannot be ruled out in this study. The detailed analyses of subgroups (Figs 4 and 5) as well as the differentiation according to elevated PAP by RHC or DE (S1 Tables) do not indicate other confounders in this study population.

### Interpretation of the survival analysis

The large study population analyzed here and the high prevalence of documented end-points offered the opportunity for detailed side-by-side statistical analysis of hemodynamic and clinical parameters for their individual prognostic value. In this unselected study population, Kaplan-Meier plots visualize a highly significant discrimination of events within the first years of follow-up defined by invasive or non-invasive as well as serological parameters (Figs 2 to 5, S1–S8 Figs). In detail, the differentiation of survival by elevated PAP measurements as shown for the unselected study population (Figs 2 and 3) was confirmed consistently in the subgroups of PH due to left heart or valvular disease (Fig 4A to 4F). In the subgroup of patients with rare cardiac diseases, consisting primarily of patients with cardiac amyloidosis with a progressive course of disease, the outcome could not be determined on elevated PAP, neither invasively by RHC nor non-invasively by DE (Fig 4G and 4H). By univariate survival analyses, the individual prognostic benefit of cardiac parameters such as PAP, RAP, NYHA FC, NT-proBNP, cTnT, LV-EF, and RV dysfunction, can be compared (Table 3). The application of multiple imputation was necessary to enable multivariate analysis of the complete dataset. For example, serological cardiac parameters were available in many, but not in all patients: NT-proBNP was measured in 643 (62%) and cTnT in 780 (75%) patients at the time of RHC. Multivariate analysis identified the independent predictors: patients with NYHA functional classes III and IV, NT-proBNP of 2,570 pg/mL and higher, cTnT of 14 ng/L and higher, LV-EF less than 40%, and RV dysfunction (Table 4). Elevated cTnT with a cut-off of 14 ng/L demonstrated a strong and independent prognostic impact in this study population. Since only a minority of patients with acute myocardial ischemia was found (n = 36; 3%), elevated cTnT may have been caused by right heart failure as well [32]. Impaired RV function was the strongest predictor for death or heart transplantation in the univariate analysis (HR 3.75, 95%CI 2.76–5.11, p<0.001, χ^2^ = 81.5, Table 3) and stayed independently predictive in multivariate analysis, whereas the hemodynamic parameters mPAP, sPAP and RAP were identified as dependent predictors. However, the analysis of multimodal diagnostic approaches that represent daily clinical routine (Fig 6) include dependent parameters as well, because they are used in corresponding settings according to current guidelines. These multimodal approaches show incremental prognostic power in this unselected study population (Fig 6). Although some diagnostic tests seem to deliver more prognostic information than others, the main issue is that concentrating on one single approach reduces information on outcome significantly.

### Translational outlook based on the study results

Although this analysis was conducted retrospectively and did not aim to evaluate therapy in patients with PH, there is a discrepancy in mortality of patients with PH that has to be discussed. Patients with precapillary PH receive focused clinical interest due to the severe prognosis if diagnosed late or left untreated. For the diagnosis of precapillary PH, a multimodal approach is mandatory, including the option of testing pulmonary arterial vasoreactivity during RHC, targeted therapy as well as follow-up examinations by specialized centers as advised by PH guidelines [7]. In spite of this devastating disease, patients show a trend to reduced mortality under treatment compared to patients with PH of other etiologies (Fig 5A) [12]. In addition to the 82 patients with precapillary PH in our analysis, a total of 265 patients with PH had an elevated PVR of 250 dyn^x^s^x^cm^-5^ or more (≥ 3.1 WU) with significantly reduced outcome compared to patients with PH and PVR less than 250 dyn^x^s^x^cm^-5^ (HR = 1.73, χ^2^ = 19.2, p<0.001, Fig 5B). Thus, the identification of patients with combined, pre- and post-capillary PH should receive increased attention in this specific hemodynamic situation, especially with a focus on women, as shown by differentiated analysis of outcome in this study (Fig 5B, S1 and S2 Figs) [33].

When comparing RHC and transthoracic echocardiography from a technical point of view, RHC remains the reference method for measuring pressures and defining pulmonary hypertension. However, cardiac ventriculography was largely abandoned for the assessment of RV function and thus, RHC is limited to deliver hemodynamic information for calculation of cardiac output and ventricular performance. On the other hand, DE is able to assess hemodynamics only indirectly, but with good correlation to invasive measurements in experienced hands [8]. The prognostic benefit of transthoracic echocardiography as demonstrated in this study population (Fig 6) is very likely based on the additional information delivered by the method: a standard echocardiographic examination includes LV function, valvular status, hemodynamic assessment and, as mentioned previously, important information on RV function. Although the examiner-dependence of echocardiography has to be kept in mind, a standardized transthoracic examination can deliver a comprehensive view of cardiopulmonary function beyond identification of pulmonary hypertension.

The results of this study show that the non-invasive detection of elevated PAP at rest has prognostic relevance, comparable to RHC measurements, in particular considering patients with chronic left heart failure. The detection of sPAP of 36 mm Hg and higher should trigger further investigation on cardiopulmonary disorders. Our analyses confirm previous clinical experience on a detailed statistical basis as well as the application of transthoracic DE as first-line diagnostic tool for patients with suspected pulmonary hypertension.

## Limitations

This study is based on retrospective and monocentric data. Furthermore, the selection of patients who received a RHC according to clinical indication and current guidelines, as well as presentation to a tertiary cardiological department may bias the severity of cardiopulmonary disease and heart failure in this study population. Multiple imputation algorithms had to be applied for multivariate analyses of the whole study population. Assessment of RV function was analyzed as documented in the echocardiographic report without quantitative re-assessment. Only a minority of the datasets included quantification of RV function by surrogate parameters.

## Conclusions

Elevated pulmonary arterial pressure assessed by Doppler echocardiography has similar predictive value regard to patient survival or need for HTx as compared with measurements by right heart catheterization. Assessments of functional capacity, serological biomarkers and transthoracic echocardiography add significant predictive information on outcome in this large and unselected study population.

## Supporting information

S1 Fig Kaplan-Meier curves for survival of patients with or without PH, differentiated by sex (a:male, b:female). Abbreviations: PH pulmonary hypertension, HR hazard ratio, 95%CI 95% confidence interval.
(PDF)

S2 Fig Kaplan-Meier curves for survival of patients with PH due to LHD or precapillary PH, differentiated by sex (a:male, b:female). Abbreviations: PH pulmonary hypertension, LHD left heart disease, HR hazard ratio, 95%CI 95% confidence interval.
(PDF)

S3 Fig **Kaplan-Meier curves for survival according to sex (a) and age (b) of patients.** Optimal cut-off of age for dichotomous analysis was determined by ROC analysis. Abbreviations: HR hazard ratio, 95%CI 95% confidence interval, ns not significant, ROC receiver-operator characteristics.
(PDF)

S4 Fig Kaplan-Meier curves for survival of patients according to clinical assessment. Optimal cut-off for dichotomous analysis was determined by ROC analysis. Abbreviations: NYHA FC New York Heart Association functional class, HR hazard ratio, 95%CI 95% confidence interval, ROC receiver-operator characteristics.
(PDF)

S5 Fig **Kaplan-Meier curves for survival according to serological cardiac parameters NT-proBNP (a), cTnT (b), and renal function (c).** Optimal cut-off for dichotomous analysis was determined by ROC analyses. Abbreviations: NT-proBNP N-terminal pro brain natriuretic peptide, cTnT cardiac troponine T, eGFR estimated glomerular filtration rate, HR hazard ratio, 95%CI 95% confidence interval, ROC receiver-operator characteristics.
(PDF)

S6 Fig **Kaplan-Meier curves for survival according to right ventricular dysfunction (a) or dilation (b) as defined by 2D echocardiography.** Optimal cut-off for dichotomous analysis was determined by ROC analyses. Abbreviations: RVEDD right ventricular end-diastolic diameter, HR hazard ratio, 95%CI 95% confidence interval, ROC receiver-operator characteristics.
(PDF)

S7 Fig **Kaplan-Meier curves for survival according to left ventricular function by CI (a) and LV-EF (b).** Optimal cut-off for dichotomous analysis was determined by ROC analyses. Abbreviations: CI cardiac index, LV-EF left ventricular ejection fraction, HR hazard ratio, 95%CI 95% confidence interval, ROC receiver-operator characteristics.
(PDF)

S8 Fig **Kaplan-Meier curves for survival in dependence of diastolic function by RAP measured invasively (a) or non-invasive (b), and invasively measured LVEDP (c).** Optimal cut-off for dichotomous analysis was determined by ROC analyses. Abbreviations: RAP right atrial pressure, LVEDP LV end-diastolic pressure, HR hazard ratio, 95%CI 95% confidence interval, ROC receiver-operator characteristics.
(PDF)

S1 Tables **Clinical characteristics of the study population and patients lost to follow-up (a), as well as study population stratified by elevated mPAP measured invasively by RHC (b), and stratified by elevated sPAP assessed non-invasively by DE (c).** Abbreviations: HTx heart transplantation, BMI body mass index, BSA body surface area, NYHA New York Heart Association, eGFR estimated glomerular filtration rate, NT-proBNP N-terminal pro brain natriuretic peptide, cTnT cardiac troponin T, CMP cardiomyopathy, IHD Ischemic heart disease, PH pulmonary hypertension, ns not significant.
(PDF)

## Abbreviations

2D: two dimensional
CI: cardiac index
CMP: cardiomyopathy
cTnT: cardiac troponin T
DE: Doppler echocardiography
eGFR: estimated glomerular filtration rate
HTx: heart transplantation
LHD: left heart disease
LV-EDP: left ventricular end-diastolic pressure
LV-EF: left ventricular ejection fraction
PAH: pulmonary arterial hypertension
PAP: pulmonary arterial pressure
PCWP: pulmonary capillary wedge pressure
PH: pulmonary hypertension
PVR: pulmonary vascular resistance
RAP: right atrial pressure
RHC: right heart catheterization
RV: right ventricular
RV-EDD: right ventricular end-diastolic diameter
TRV: tricuspid regurgitation velocity
