# Supplementary material for: Prognostic relevance of elevated pulmonary arterial pressure assessed non-invasively: Analysis in a large patient cohort with invasive measurements in near temporal proximity
Source: PLoS One. 2018 Jan 19;13(1):e0191206. doi: 10.1371/journal.pone.0191206 (PMC5774714; doi:10.1371/journal.pone.0191206)
Supplement: S1 Fig — Abbreviations: PH pulmonary hypertension, HR hazard ratio, 95%CI 95% confidence interval. (PDF) [file pone.0191206.s001.pdf]

S1 Fig. Kaplan-Meier curves for survival of patients with or without PH, differentiated by sex (a:male, b:female). Abbreviations: PH pulmonary hypertension, HR hazard ratio, 95%CI 95% confidence interval

A

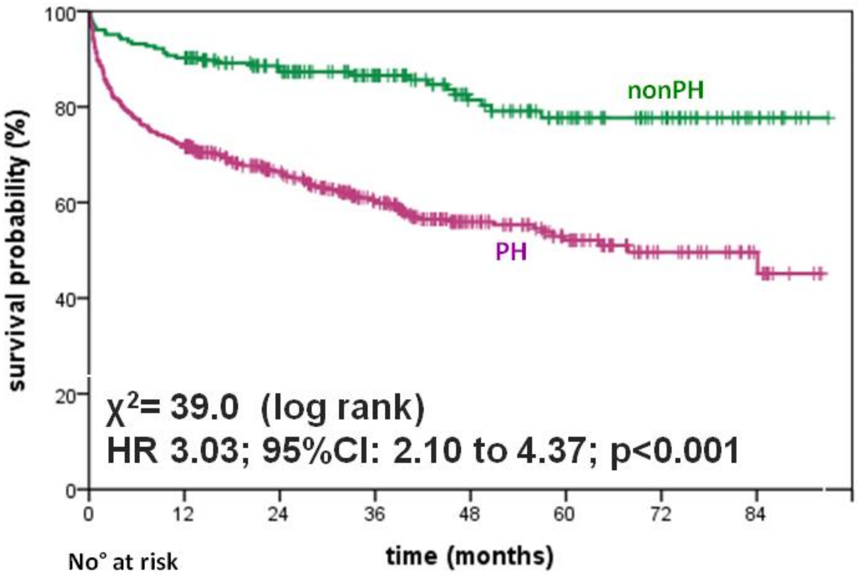

No° at risk

group 0: male nonPH

205	185	140	106	70	50	33	12

group 1: male PH

455	329	234	158	93	60	26	11

B

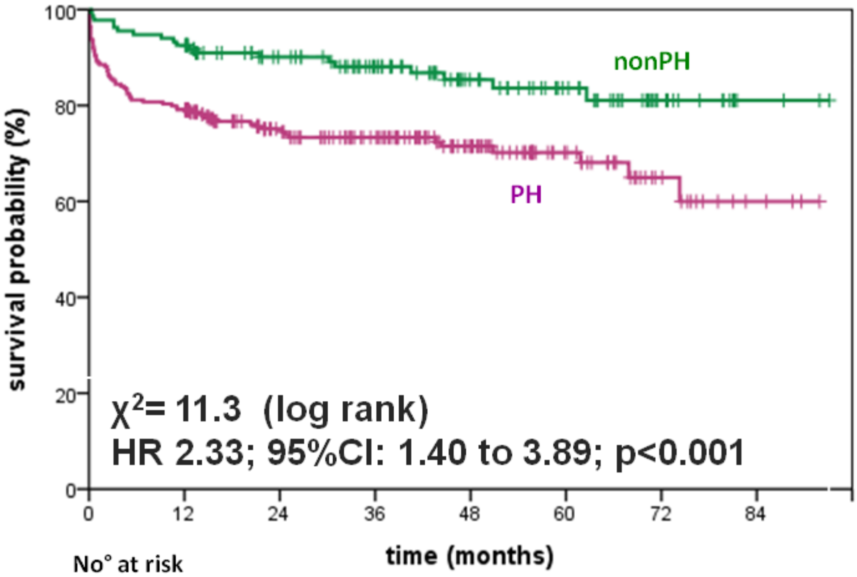

No° at risk

group 0: female nonPH

134	124	97	77	51	36	15	3

group 1: female PH

244	193	130	104	68	35	14	4
