# Supplementary material for: Prognostic relevance of elevated pulmonary arterial pressure assessed non-invasively: Analysis in a large patient cohort with invasive measurements in near temporal proximity
Source: PLoS One. 2018 Jan 19;13(1):e0191206. doi: 10.1371/journal.pone.0191206 (PMC5774714; doi:10.1371/journal.pone.0191206)
Supplement: S2 Fig — Abbreviations: PH pulmonary hypertension, LHD left heart disease, HR hazard ratio, 95%CI 95% confidence interval. (PDF) [file pone.0191206.s002.pdf]

S2 Fig. Kaplan-Meier curves for survival of patients with PH due to LHD or precapillary PH, differentiated by sex (a:male, b:female). Abbreviations: PH pulmonary hypertension, LHD left heart disease, HR hazard ratio, 95%CI 95% confidence interval

A

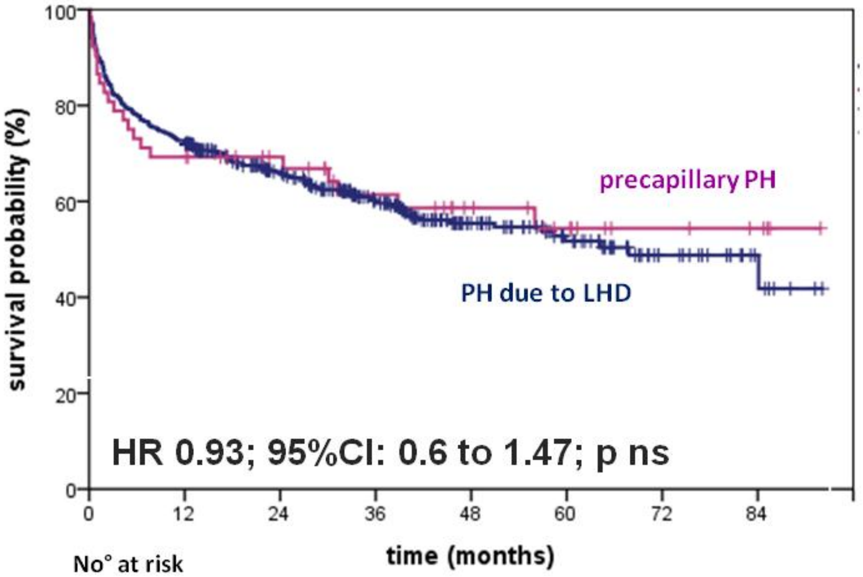

No° at risk

group 0: male, PH due to LHD							
403	293	205	136	77	49	20	7
group 1: male, precapillary PH							
52	36	29	22	16	11	6	4

B

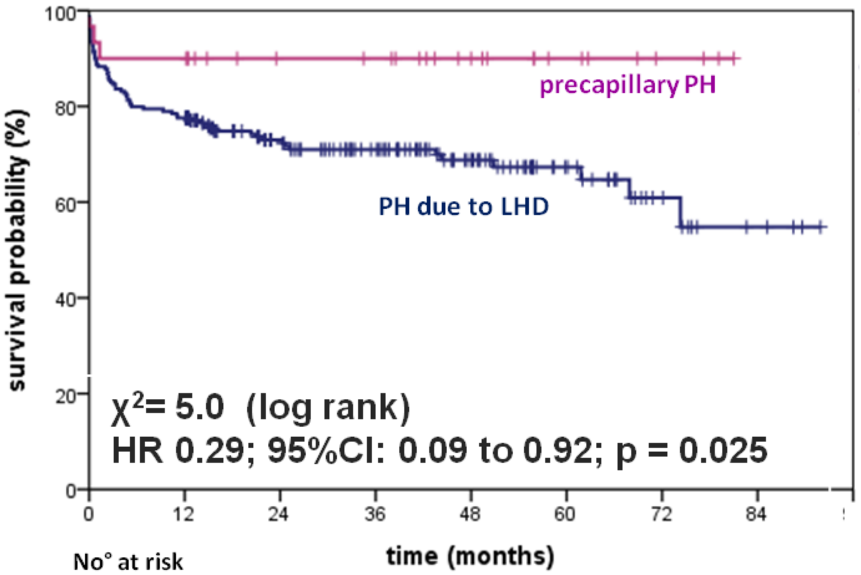

No° at risk

group 0: female, PH due to LHD							
214	166	110	85	55	28	11	4
group 1: female, precapillary PH							
30	27	20	19	13	7	3	-
