# Supplementary material for: Prognostic relevance of elevated pulmonary arterial pressure assessed non-invasively: Analysis in a large patient cohort with invasive measurements in near temporal proximity
Source: PLoS One. 2018 Jan 19;13(1):e0191206. doi: 10.1371/journal.pone.0191206 (PMC5774714; doi:10.1371/journal.pone.0191206)
Supplement: S3 Fig — Kaplan-Meier curves for survival according to sex (a) and age (b) of patients. Optimal cut-off of age for dichotomous analysis was determined by ROC analysis. Abbreviations: HR hazard ratio, 95%CI 95% confidence interval, ns not significant, ROC receiver-operator characteristics. (PDF) [file pone.0191206.s003.pdf]

S3 Fig. Kaplan-Meier curves for survival according to sex (a) and age (b) of patients.
Optimal cut-off of age for dichotomous analysis was determined by ROC analysis. Abbreviations: HR hazard ratio, 95%CI 95% confidence interval, ns not significant, ROC receiver-operator characteristics

A

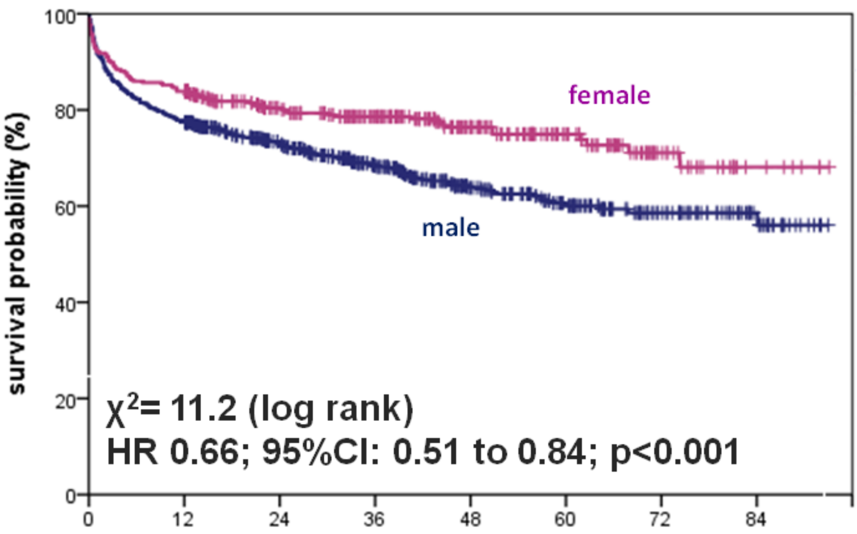

No° at risk

group 0: men

660	514	374	264	163	110	59	23

group 1: women

378	317	227	181	119	71	29	7

B

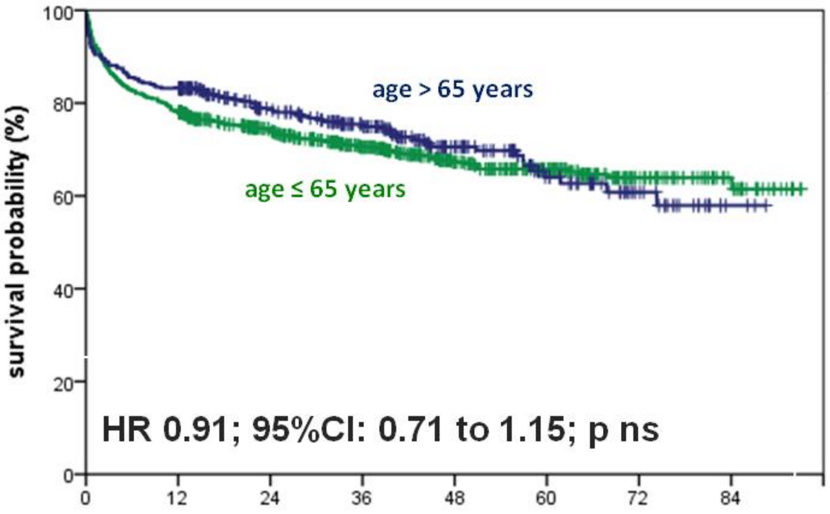

No° at risk

group 0: age < 65 years

693	544	403	297	192	131	64	27

group 1: age ≥ 65 years

345	287	198	148	90	50	24	3
