# Supplementary material for: Prognostic relevance of elevated pulmonary arterial pressure assessed non-invasively: Analysis in a large patient cohort with invasive measurements in near temporal proximity
Source: PLoS One. 2018 Jan 19;13(1):e0191206. doi: 10.1371/journal.pone.0191206 (PMC5774714; doi:10.1371/journal.pone.0191206)
Supplement: S4 Fig — Optimal cut-off for dichotomous analysis was determined by ROC analysis. Abbreviations: NYHA FC New York Heart Association functional class, HR hazard ratio, 95%CI 95% confidence interval, ROC receiver-operator characteristics. (PDF) [file pone.0191206.s004.pdf]

S4 Fig. Kaplan-Meier curves for survival of patients according to clinical assessment. Optimal cut-off for dichotomous analysis was determined by ROC analysis. Abbreviations: NYHA FC New York Heart Association functional class, HR hazard ratio, 95%CI 95% confidence interval, ROC receiver-operator characteristics

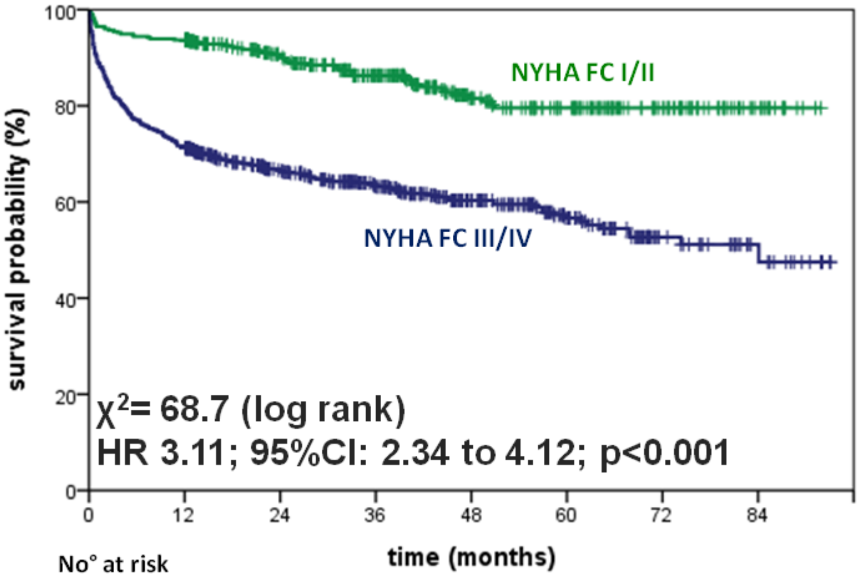

group 0: NYHA FC class I/II

395	370	282	212	129	92	53	16

group 1: NYHA FC class III/IV

643	461	319	233	153	89	35	14
