# Supplementary material for: Prognostic relevance of elevated pulmonary arterial pressure assessed non-invasively: Analysis in a large patient cohort with invasive measurements in near temporal proximity
Source: PLoS One. 2018 Jan 19;13(1):e0191206. doi: 10.1371/journal.pone.0191206 (PMC5774714; doi:10.1371/journal.pone.0191206)
Supplement: S5 Fig — Kaplan-Meier curves for survival according to serological cardiac parameters NT-proBNP (a), cTnT (b), and renal function (c). Optimal cut-off for dichotomous analysis was determined by ROC analyses. Abbreviations: NT-proBNP N-terminal pro brain natriuretic peptide, cTnT cardiac troponine T, eGFR estimated glomerular filtration rate, HR hazard ratio, 95%CI 95% confidence interval, ROC receiver-operator characteristics. (PDF) [file pone.0191206.s005.pdf]

S5 Fig. Kaplan-Meier curves for survival according to serological cardiac parameters NT-proBNP (a), cTnT (b), and renal function (c). Optimal cut-off for dichotomous analysis was determined by ROC analyses. Abbreviations: NT-proBNP N-terminal pro brain natriuretic peptide, cTnT cardiac troponine T, eGFR estimated glomerular filtration rate, HR hazard ratio, 95%CI 95% confidence interval, ROC receiver-operator characteristics

A

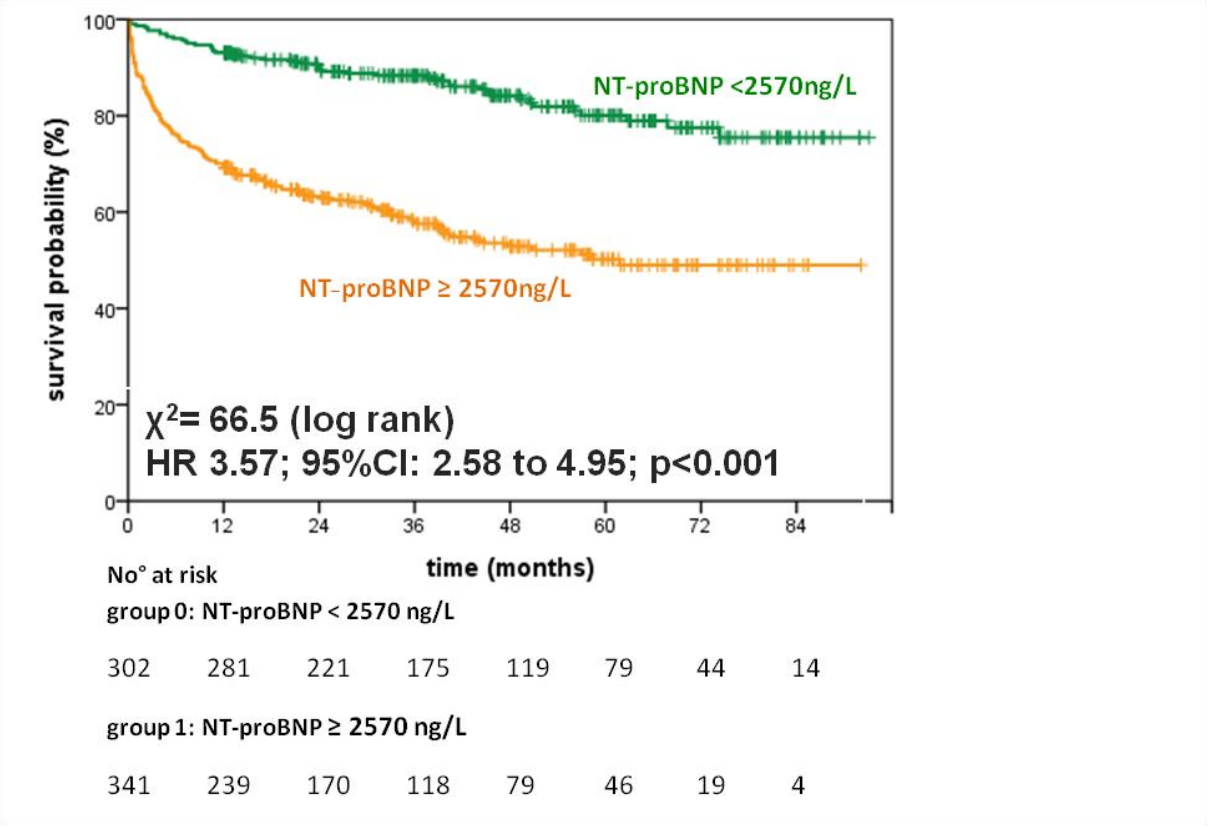

B

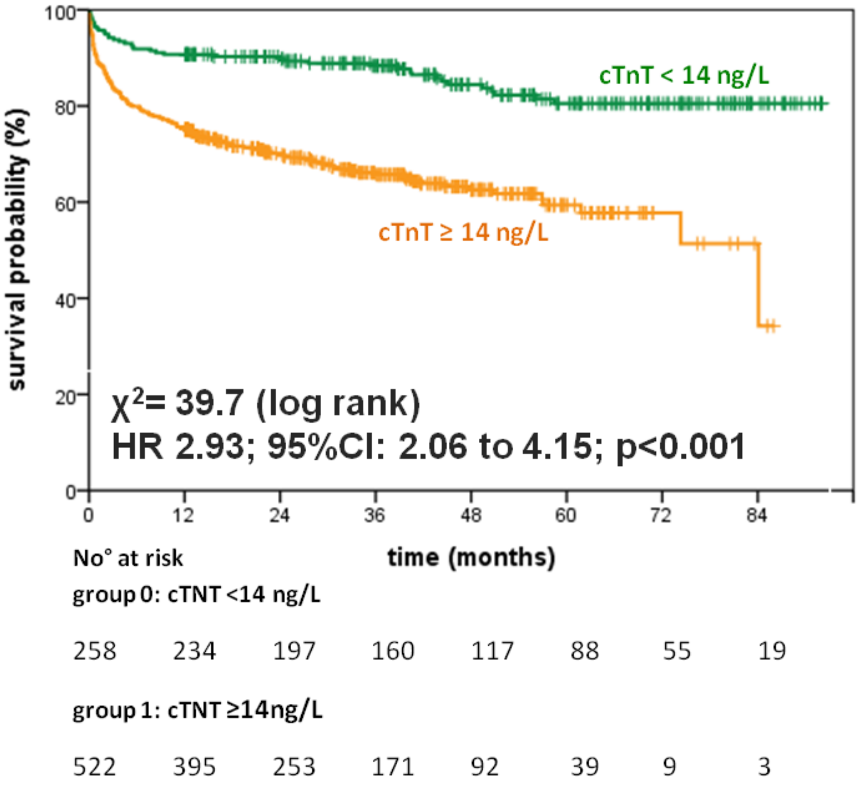

C

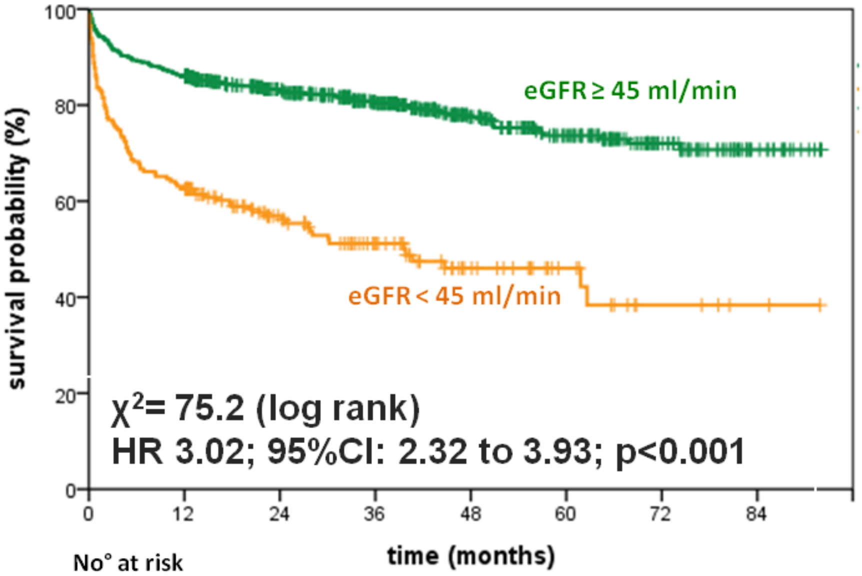

No° at risk

group 0: eGFR ≥ 45ml/min

657	567	412	300	186	122	61	17

group 1: eGFR < 45 ml/min

195	123	76	49	26	14	5	2
