# Supplementary material for: Prognostic relevance of elevated pulmonary arterial pressure assessed non-invasively: Analysis in a large patient cohort with invasive measurements in near temporal proximity
Source: PLoS One. 2018 Jan 19;13(1):e0191206. doi: 10.1371/journal.pone.0191206 (PMC5774714; doi:10.1371/journal.pone.0191206)
Supplement: S6 Fig — Kaplan-Meier curves for survival according to right ventricular dysfunction (a) or dilation (b) as defined by 2D echocardiography. Optimal cut-off for dichotomous analysis was determined by ROC analyses. Abbreviations: RVEDD right ventricular end-diastolic diameter, HR hazard ratio, 95%CI 95% confidence interval, ROC receiver-operator characteristics. (PDF) [file pone.0191206.s006.pdf]

S6 Fig. Kaplan-Meier curves for survival according to right ventricular dysfunction (a) or dilation (b) as defined by 2D echocardiography. Optimal cut-off for dichotomous analysis was determined by ROC analyses. Abbreviations: RVEDD right ventricular end-diastolic diameter, HR hazard ratio, 95%CI 95% confidence interval, ROC receiver-operator characteristics

A

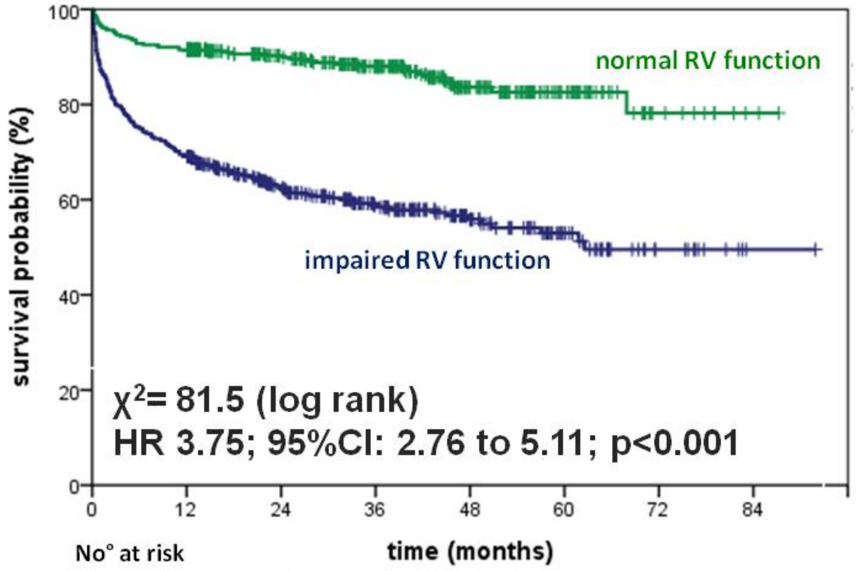

No° at risk

group 0: normal RV function (echo)							
389	356	262	191	96	43	10	2
group 1: impaired RV function (echo)							
456	317	201	130	72	35	11	1

B

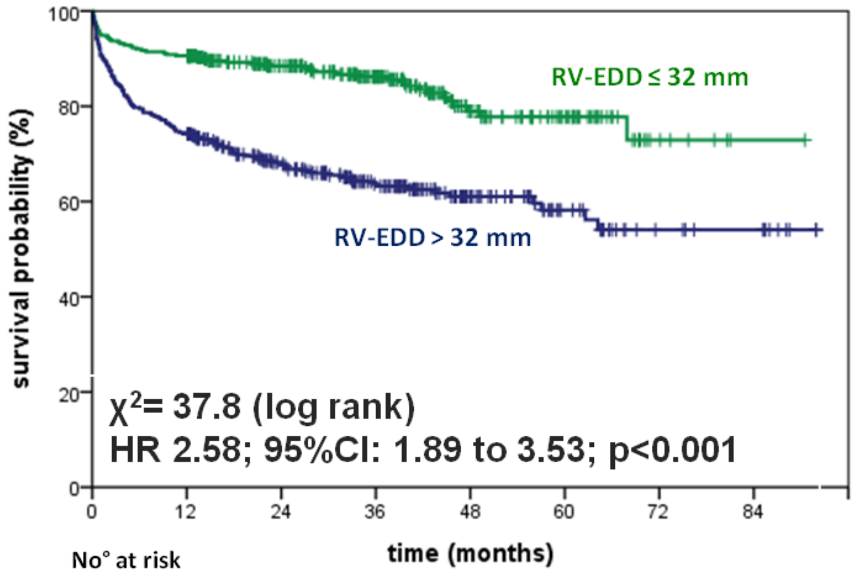

No° at risk

group 0: RV-EDD ≤ 32 mm							
350	317	220	154	72	38	7	1
group 1: RV-EDD > 32 mm							
380	283	187	124	66	31	13	10
