# Supplementary material for: Prognostic relevance of elevated pulmonary arterial pressure assessed non-invasively: Analysis in a large patient cohort with invasive measurements in near temporal proximity
Source: PLoS One. 2018 Jan 19;13(1):e0191206. doi: 10.1371/journal.pone.0191206 (PMC5774714; doi:10.1371/journal.pone.0191206)
Supplement: S7 Fig — Kaplan-Meier curves for survival according to left ventricular function by CI (a) and LV-EF (b). Optimal cut-off for dichotomous analysis was determined by ROC analyses. Abbreviations: CI cardiac index, LV-EF left ventricular ejection fraction, HR hazard ratio, 95%CI 95% confidence interval, ROC receiver-operator characteristics. (PDF) [file pone.0191206.s007.pdf]

S7 Fig. Kaplan-Meier curves for survival according to left ventricular function by CI (a) and LV-EF (b). Optimal cut-off for dichotomous analysis was determined by ROC analyses. Abbreviations: CI cardiac index, LV-EF left ventricular ejection fraction, HR hazard ratio, 95%CI 95% confidence interval, ROC receiver-operator characteristics

A

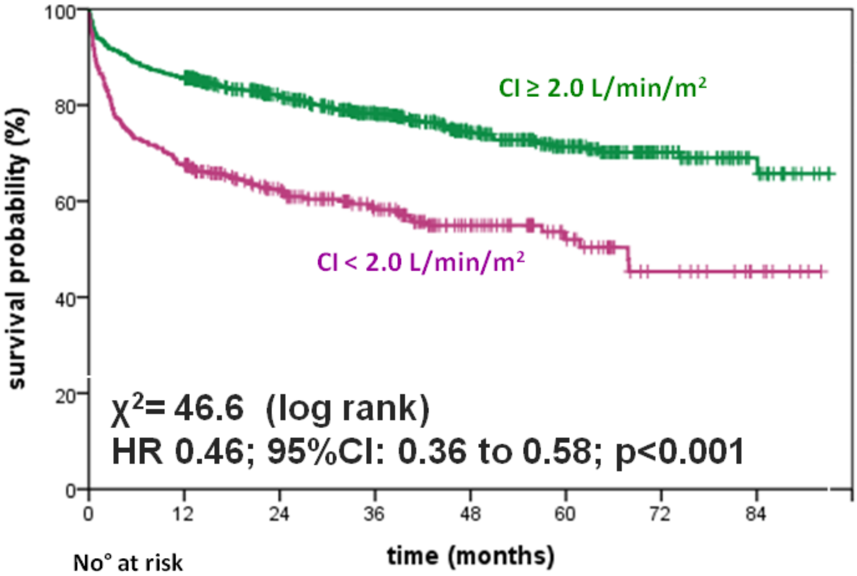

No° at risk

group 0: CI ≥ 2.0 L/min/m²							
688	591	431	324	210	138	69	21
group 1: CI < 2.0 L/min/m²							
311	211	147	102	60	34	15	7

B

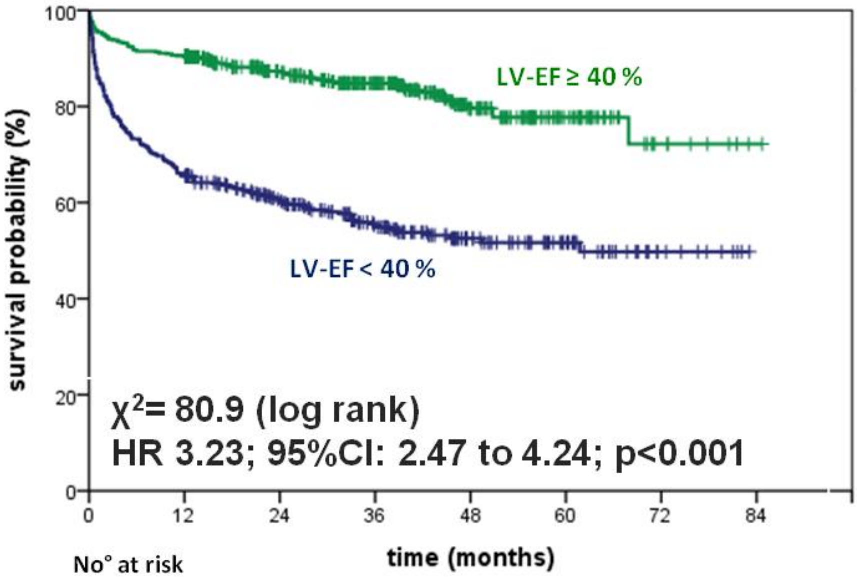

No° at risk

group 0: LV-EF ≥ 40 %							
483	437	294	209	103	41	8	1
group 1: LV-EF < 40 %							
366	242	173	114	64	35	9	-
