# Supplementary material for: Prognostic relevance of elevated pulmonary arterial pressure assessed non-invasively: Analysis in a large patient cohort with invasive measurements in near temporal proximity
Source: PLoS One. 2018 Jan 19;13(1):e0191206. doi: 10.1371/journal.pone.0191206 (PMC5774714; doi:10.1371/journal.pone.0191206)
Supplement: S8 Fig — Kaplan-Meier curves for survival in dependence of diastolic function by RAP measured invasively (a) or non-invasive (b), and invasively measured LVEDP (c). Optimal cut-off for dichotomous analysis was determined by ROC analyses. Abbreviations: RAP right atrial pressure, LVEDP LV end-diastolic pressure, HR hazard ratio, 95%CI 95% confidence interval, ROC receiver-operator characteristics. (PDF) [file pone.0191206.s008.pdf]

S8 Fig. Kaplan-Meier curves for survival in dependence of diastolic function by RAP measured invasively (a) or non-invasive (b), and invasively measured LVEDP (c). Optimal cut-off for dichotomous analysis was determined by ROC analyses. Abbreviations: RAP right atrial pressure, LVEDP LV end-diastolic pressure, HR hazard ratio, 95%CI 95% confidence interval, ROC receiver-operator characteristics

A

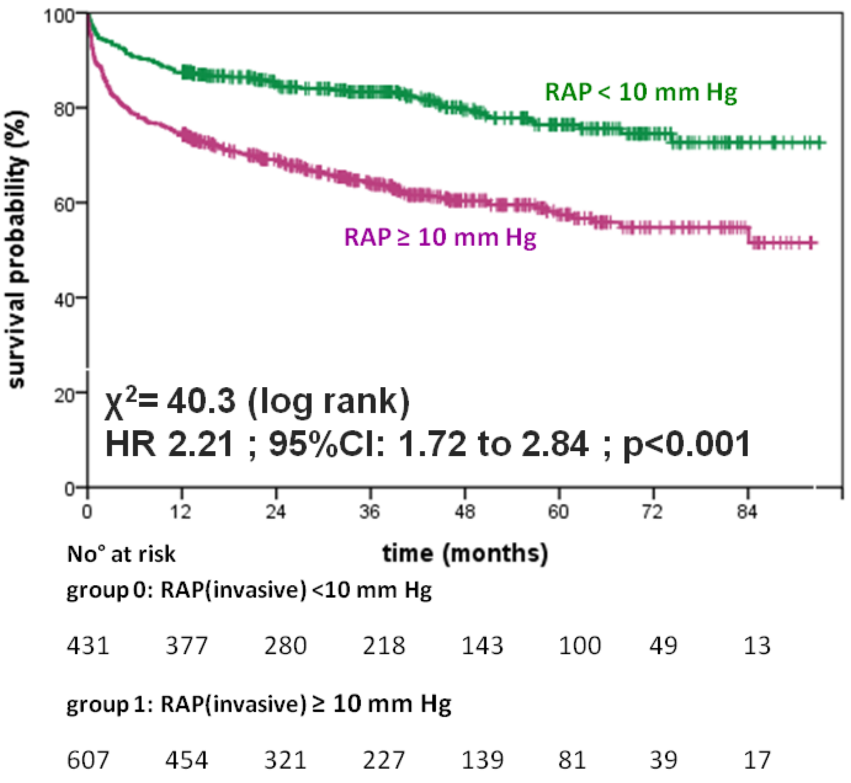

B

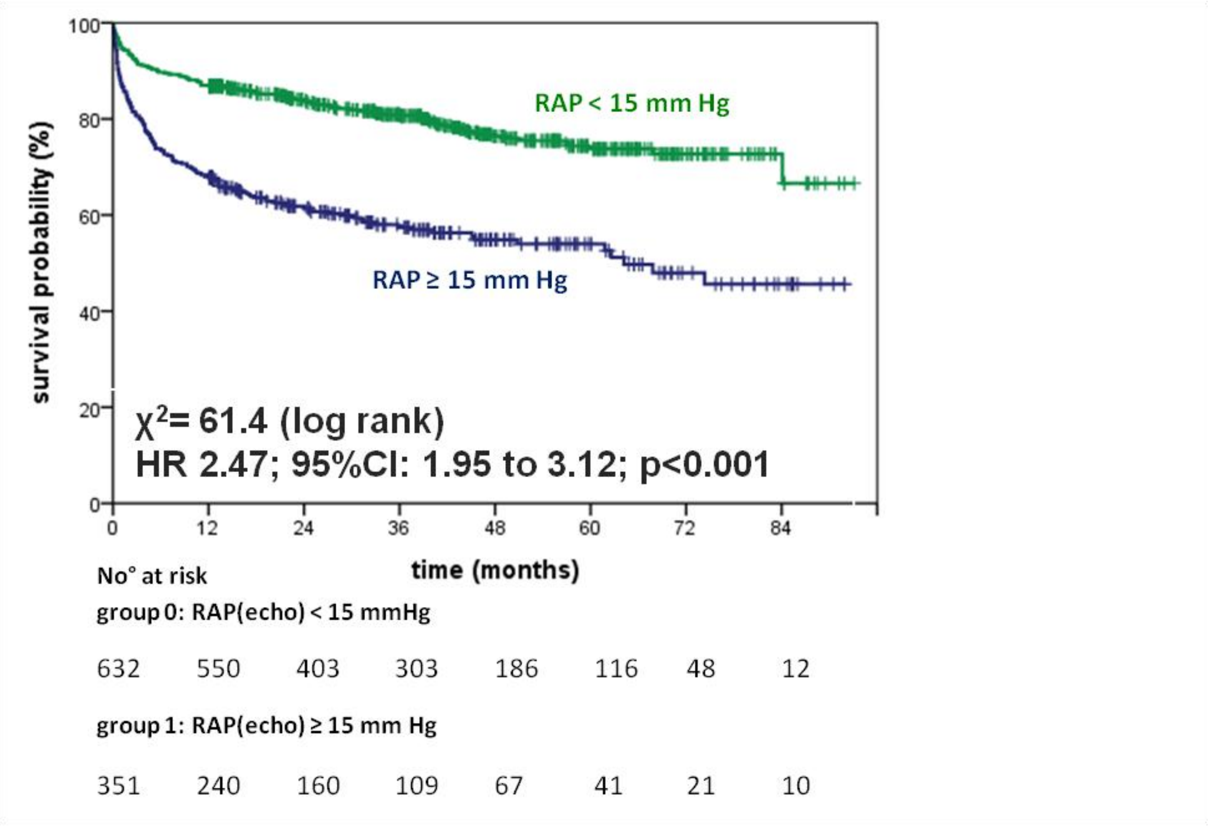

C

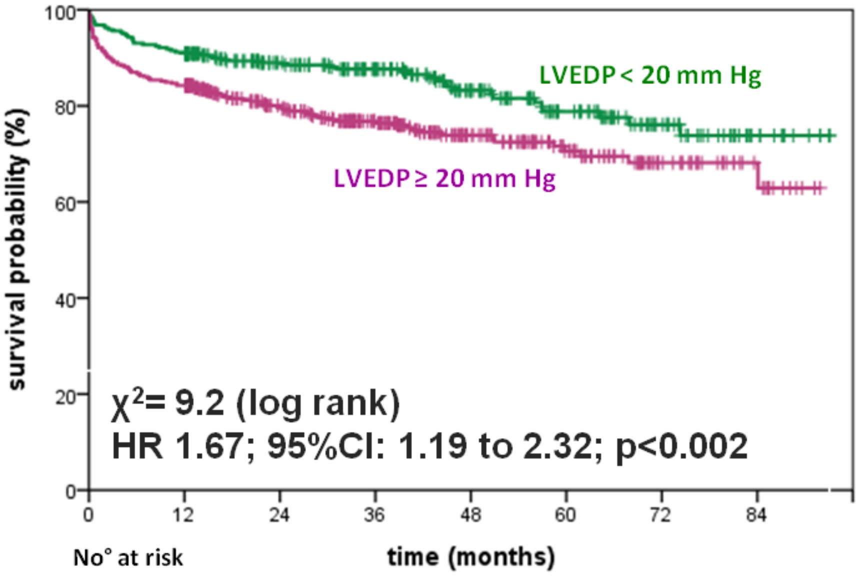

No° at risk

group 0: LVEDP < 20 mm Hg

317	289	209	168	114	75	40	12

group 1: LVEDP ≥ 20 mm Hg

431	363	261	186	114	72	34	13
