# Supplementary material for: Prognostic relevance of elevated pulmonary arterial pressure assessed non-invasively: Analysis in a large patient cohort with invasive measurements in near temporal proximity
Source: PLoS One. 2018 Jan 19;13(1):e0191206. doi: 10.1371/journal.pone.0191206 (PMC5774714; doi:10.1371/journal.pone.0191206)
Supplement: S1 Tables — Clinical characteristics of the study population and patients lost to follow-up (a), as well as study population stratified by elevated mPAP measured invasively by RHC (b), and stratified by elevated sPAP assessed non-invasively by DE (c). Abbreviations: HTx heart transplantation, BMI body mass index, BSA body surface area, NYHA New York Heart Association, eGFR estimated glomerular filtration rate, NT-proBNP N-terminal pro brain natriuretic peptide, cTnT cardiac troponin T, CMP cardiomyopathy, IHD Ischemic heart disease, PH pulmonary hypertension, ns not significant. (PDF) [file pone.0191206.s009.pdf]

S1 Tables. Clinical characteristics of the study population and patients lost to follow-up (a), as well as study population stratified by elevated mPAP measured invasively by RHC (b), and stratified by elevated sPAP assessed non-invasively by DE (c). Abbreviations: HTx heart transplantation, BMI body mass index, BSA body surface area, NYHA New York Heart Association, eGFR estimated glomerular filtration rate, NT-proBNP N-terminal pro brain natriuretic peptide, cTnT cardiac troponin T, CMP cardiomyopathy, IHD Ischemic heart disease, PH pulmonary hypertension, ns not significant.

A. Clinical characteristics of the study population and patients lost to follow-up.

**Parameter**	**all**<br>**n = 1 038**	**lost to follow-up**<br>**n = 199**	**p-value**
Age, y	66 ± 15	70 ± 15	0.001
Males, n (%)	660 (64)	105 (53)	0.040
Height, cm	171 ± 10	169 ± 10	ns
Weight, kg	79 ± 17	79 ± 19	ns
BMI, kg/m²	27 ± 5	27 ± 5	ns
BSA, m²	1.9 ± 0.2	1.9 ± 0.2	ns
**Clinical classification, n (%)**			
NYHA functional class I	84 (8)	11 (6)	
NYHA functional class II	311 (30)	73 (36)	ns
NYHA functional class III	533 (51)	95 (48)	
NYHA functional class IV	110 (11)	20 (10)	
eGFR, mL/min/1.73m²	66 ± 27	62 ± 27	ns
NT-proBNP, pg/mL	2889 (847;6731)	2862 (1035; 6297)	ns
cTnT, ng/L	24 (10;59)	30 (12; 85)	0.045
**Indications for heart catheterization, n (%)**			
Known or suspected CMP	261 (25)	47 (24)	ns
Evaluation valve disease			
-Aortic valve disease	186 (18)	35 (17)	ns
-Mitral valve disease	82 (8)	20 (10)	ns
-Tricuspid valve disease	6 (1)	1 (1)	ns
Known or suspected IHD	315 (30)	60 (30)	ns
Known or suspected precapillary PH	82 (8)	20 (10)	ns
Other miscellaneous indications[a]	106 (10)	16 (8)	ns

[a]primarily cardiac amyloidosis

B. Study population stratified by elevated mPAP, measured invasively by RHC.

Parameter	mPAP < 25 mm Hg<br>n = 339	mPAP ≥ 25 mm Hg<br>n = 699	p-value
Age, y	65 ± 15	66 ± 15	ns
Males, n (%)	205 (60)	455 (65)	ns
Height, cm	170 ± 10	172 ± 9	ns
Weight, kg	73 ± 13	82 ± 18	<0.001
BMI, kg/m²	25 ± 4	27 ± 6	<0.001
BSA, m²	1.8 ± 0.2	1.9 ± 0.2	<0.001
**Clinical classification, n (%)**			
NYHA functional class I	58 (17)	26 (4)	<0.001
NYHA functional class II	138 (41)	173 (25)	
NYHA functional class III	126 (37)	407 (58)	
NYHA functional class IV	17 (5)	93 (13)	
eGFR, mL/min/1.73m²	74 ± 26	62 ± 27	<0.001
NT-proBNP, pg/mL	802 (278;2564)	4156 (1710; 9155)	<0.001
cTnT, ng/L	11 (10;32)	33 (15;71)	<0.001
**Indications for heart catheterization, n (%)**			
Known or suspected CMP	63 (19)	198 (28)	ns
Evaluation valve disease			
-Aortic valve disease	83 (24)	103(15)	0.026
-Mitral valve disease	31 (9)	51 (7)	ns
-Tricuspid valve disease	1 (0.2)	5 (1)	ns
Known or suspected IHD	118 (35)	197 (28)	ns
Known or suspected precapillary PH	0 (0)	82 (12)	0.001
Other miscellaneous indications[a]	43 (10)	63 (9)	ns

[a]primarily cardiac amyloidosis

C. Study population stratified by elevated sPAP, assessed non-invasively by DE.

Parameter	sPAP < 36 mm Hg n = 381	sPAP ≥ 36 mm Hg n = 657	p-value
Age, y	64 ± 15	67 ± 15	ns
Males, n (%)	250 (66)	410 (62)	ns
Height, cm	172 ± 9	171 ± 10	ns
Weight, kg	77 ± 15	81 ± 18	ns
BMI, kg/m²	26 ± 5	28 ± 6	ns
BSA, m²	1.9 ± 0.2	1.9 ± 0.2	ns
**Clinical classification, n (%)**			
NYHA functional class I	64 (17)	20 (3)	
NYHA functional class II	146 (38)	165 (25)	p<0.001
NYHA functional class III	145 (38)	388 (59)	
NYHA functional class IV	26 (7)	84 (13)	
eGFR, mL/min/1.73m²	72 ± 26	63 ± 27	p<0.001
NT-proBNP, pg/mL	985 (321;3342)	4162 (1681;9106)	p<0.001
cTnT, ng/L	14 (10;40)	30 (14;67)	p<0.001
**Indications for heart catheterization, n (%)**			
Known or suspected CMP	89 (24)	172 (26)	ns
Evaluation valve disease			
-Aortic valve disease	84 (22)	102 (15)	ns
-Mitral valve disease	39 (10)	43 (7)	ns
-Tricuspid valve disease	0 (0)	6 (1)	ns
Known or suspected IHD	126 (33)	189 (29)	ns
Known or suspected precapillary PH	9 (2)	73 (11)	0.01
Other miscellaneous indications[a]	34 (9)	72 (11)	ns

[a]primarily cardiac amyloidosis
